# Supplementary figures and images for: Coupling spectral imaging and laboratory analyses to digitally map sediment parameters and stratigraphic layers in Yeha, Ethiopia
Source: PLoS One. 2020 Sep 11;15(9):e0238894. doi: 10.1371/journal.pone.0238894 (PMC7485874; doi:10.1371/journal.pone.0238894)

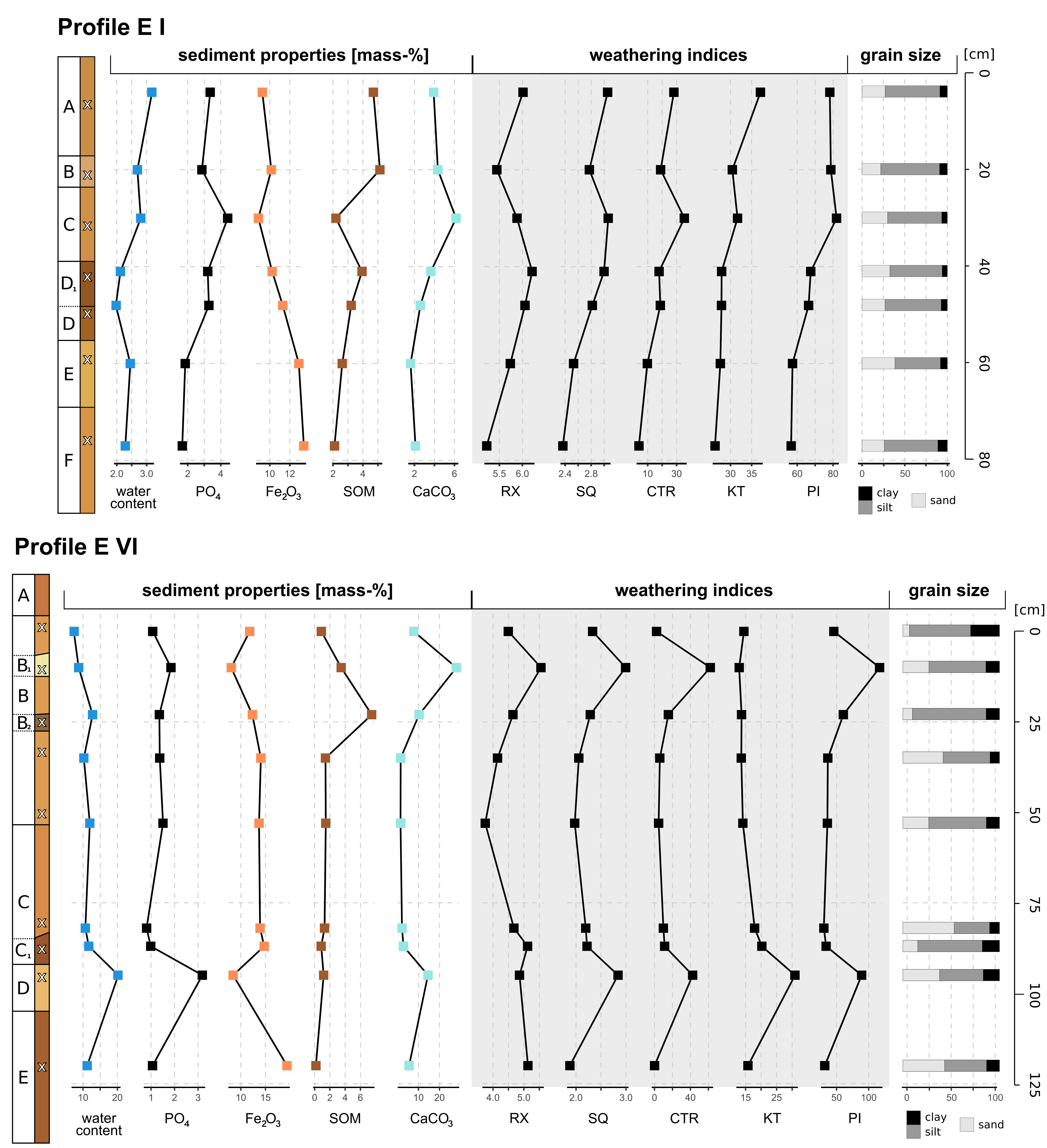

Supplement: S1 Fig — Water content of the sampled material was determined gravimetrically. Soil organic matter was calculated from quantitative measurements of total carbon (LECO TruspecCHN) and total inorganic carbon (Woesthoff Carmhograph C-16). Particle size distribution was measured using a laser diffraction particle size analyser (Beckmann-Coulter LS13 320). Element contents were measured using a portable energy-dispersive X-ray fluorescence spectrometer (Thermo Scientific Niton XL3t) and a inductively coupled plasma optical emission spectrometer (PerkinElmer Optima 2100 DV). Mineral composition was measured using an X-ray powder diffractometer (Rigaku MiniFlex 600). Weathering indices Ca-Ti-ratio (CTR), Ruxton-ratio (RX), Sesquioxide-ratio (SQ), K-Ti-ratio (KT) and Parker’s Index (PI) were calculated. The methodology is described in more detail in the Materials and methods section. (PNG) [file pone.0238894.s001.png]

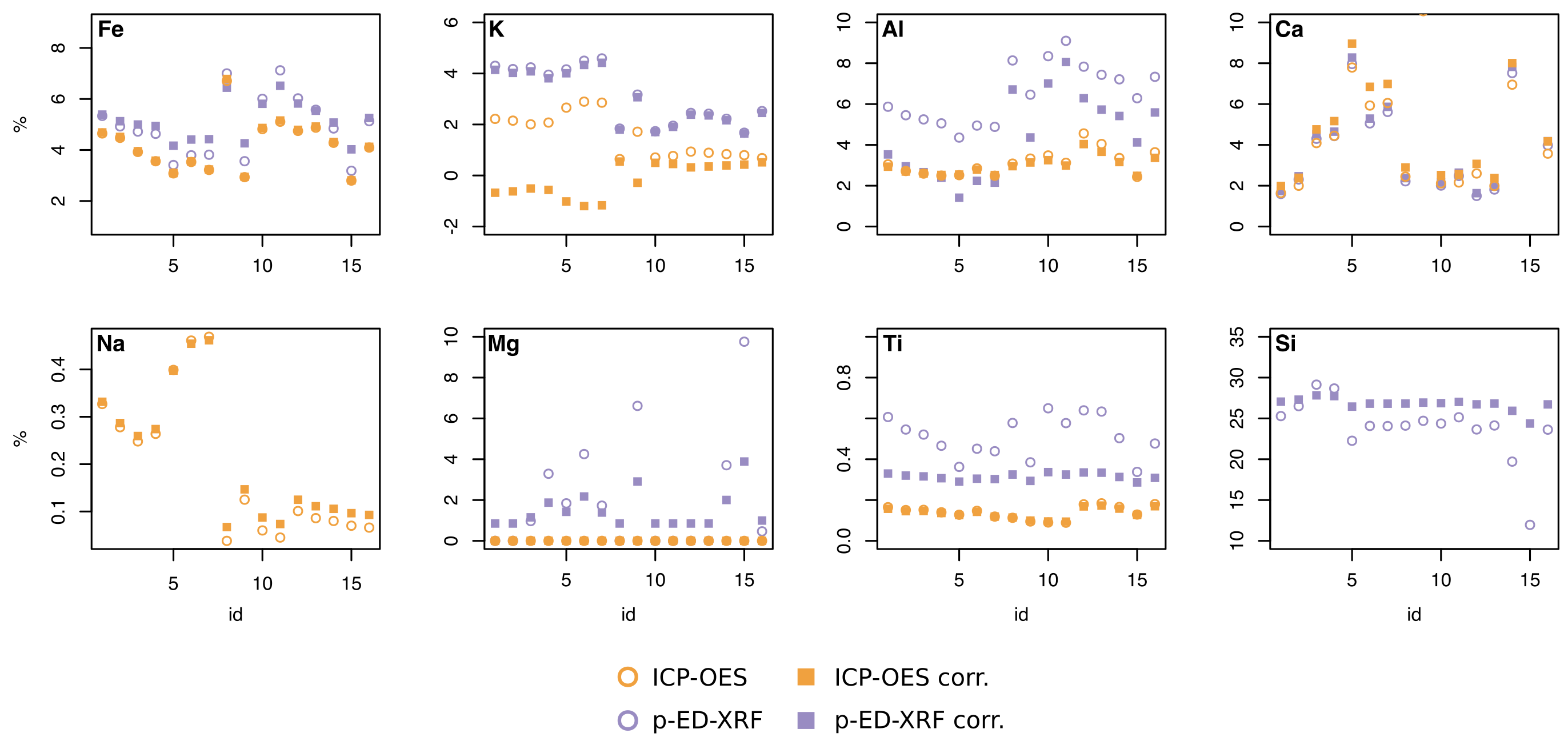

Supplement: S1 File — Files available at: https://doi.org/10.5281/zenodo.3906210. (ZIP) [file pone.0238894.s002.zip › S2_File/XRF_ICP_comparison.png]

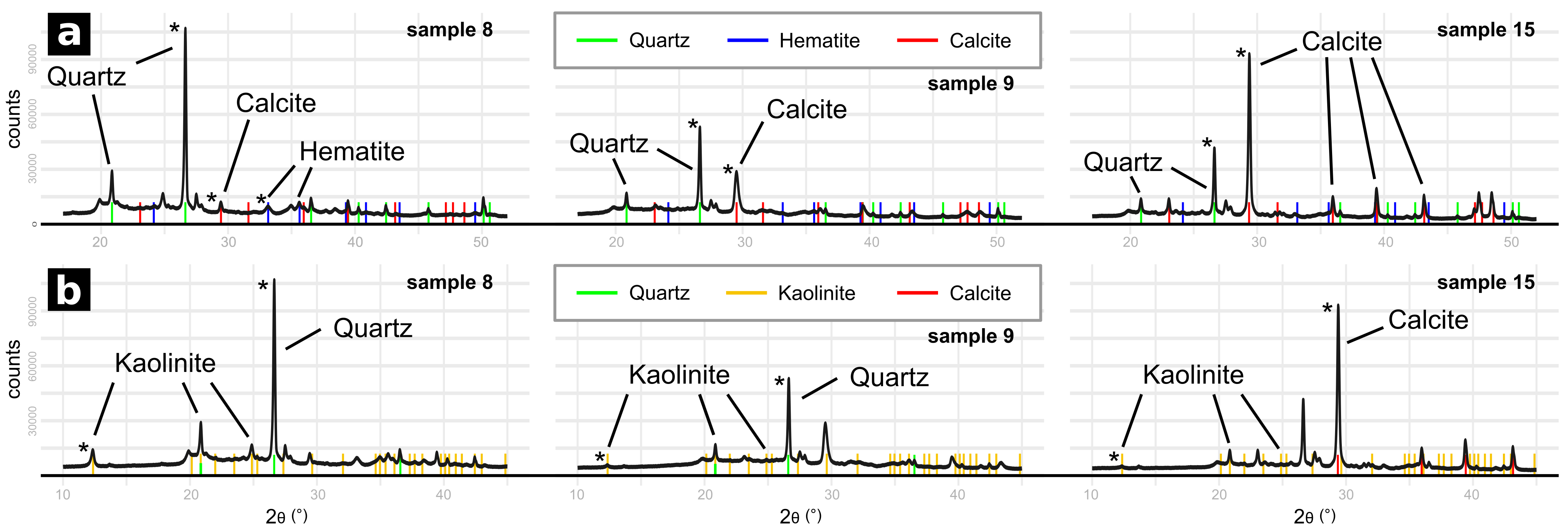

Supplement: S1 File — Files available at: https://doi.org/10.5281/zenodo.3906210. (ZIP) [file pone.0238894.s002.zip › S2_File/XRD_peaks.png]
