# Supplementary material for: Coupling spectral imaging and laboratory analyses to digitally map sediment parameters and stratigraphic layers in Yeha, Ethiopia
Source: PLoS One. 2020 Sep 11;15(9):e0238894. doi: 10.1371/journal.pone.0238894 (PMC7485874; doi:10.1371/journal.pone.0238894)
Supplement: Attachment — Submitted filename: Response to Reviewers.pdf [file pone.0238894.s005.pdf]

PONE-D-20-19503

Coupling spectral imaging and laboratory analyses to digitally map sediment parameters and stratigraphic layers in Yeha, Ethiopia

PLOS ONE

Vincent Haburaj, 4 August 2020

Original comment of Reviewer 1:

This contribution presents a relevant study that was performed in a satisfactory manner. The manuscript is written in a transparent fashion, with its discussion and conclusions sufficiently supported by the data, argumentations, and literature. The descriptions of the materials and methods sections are exhaustive. The statistical analyses are suited to the types of data and performed satisfactorily. Inconsistent or unexpected results are discussed in the text. The figures are of high quality and appropriate in the light of the presented evidence. The data underlying the manuscript are available either within the manuscript or as supplementary information. The recommendations for future studies are supported by the rest of the manuscript.

There are a few minor grammatical and typographical errors and, while they do not interfere with the legibility of the manuscript, I would advise the authors to perform one additional round of revision, preferably by a native speaker or equivalent. In the Profile descriptions section, there is some inconsistency (in the main text) on the use of profile names, e.g. E I versus EI, etc. The same goes for instance for the capitalisation of hematite/Hematite. Regarding archaeological terminology, I recommend to replace the term 'cultural layers' with the less interpretation-heavy term 'anthropogenic deposits' or 'anthropogenic layers' (or possibly 'occupation deposits' if preferred), and the uses of the word 'cultural', for instance in conjunction with 'activity' or 'remains', by 'anthropogenic' as well in order to reflect more recent internationally accepted terminology.

Answer to Reviewer 1:

Thank you very much for reviewing our paper. We corrected the mentioned grammatical and typographical errors. The named passages were adjusted accordingly. Profile names are now consistent throughout the text. As suggested, the term ‘anthropogenic layers’ is now used instead of ‘cultural layers’. Furthermore, proof-reading was carried out by a native speaker and the text was changed in several passages to improve legibility and consistency.

Original comment of Reviewer 2:

This is an outstanding article that presents an innovative solution to mapping stratigraphy in archaeological sites using a robust quantitative approach based on imaging. I recommend it unreservedly for publication, other than some instances of awkward phrasing, which are

particularly obvious in the early parts of the manuscript (and are highlighted in the attached marked up version of the manuscript).

Answer to Reviewer 2:

Thank you very much for reviewing our paper and highlighting several language errors. The mentioned passages were adjusted accordingly. Furthermore, proof-reading was carried out by a native speaker and the text was changed in several passages to improve legibility and consistency.

Original comment of Editor:

In addition to the reviewers' comments, I have one additional remark, which relates to your conclusion. In the final paragraph, you state that 'complementing the presented approach with unsupervised classification algorithms' would allow mapping stratigraphic layers semi-automatically. I think this statement needs further clarification (and for instance be moved to the discussion), or should be removed. It is unclear to me if you propose replacing supervised classification methods with unsupervised ones, or combining the current methodology with unsupervised approaches. If the former is the case, you do not present enough information in the paper to back this statement (particularly as you show under 'reproducibility' that the way in which training data are integrated (single profile/multi profile) affects the end result. How you would leap from this reduced accuracy to successful implementation of unsupervised approaches is unclear to me.
If, on the other hand, you propose a integrating unsupervised learning approaches into the current procedure, you should make explicit how you envision this precisely. In this case, and should you wish to elaborate on this issue, I suggest moving this part to the discussion. As this seems more a sidenote to your well presented methodology and results, it may be, in this case, perhaps be more suited to remove the statement.
Additionally, for Figure S1 (appendix 1) please provide information on the methodology to obtain the presented results in appendix 1. As this is separate from the text, and referred to under 'profile description' before the method section, this is a bit confusion. I suggest adding this information in the figure caption (where you can also refer to the method section).

Answer to Editor:

Thank you for considering our paper for publication. As mentioned above, the text was proof-read and corrected so that all problems regarding grammatical and typological errors should be solved.
We added a short description of the methods used to produce Figure S1 to the caption of the figure (see manuscript). Thank you for pointing that out.
The mentioned passage in the final paragraph of the conclusions was removed from the text, since it was just a sidenote referring to a possible integration of unsupervised learning approaches into the current procedure. As you pointed out, further elaboration on the topic would probably shift the focus of the conclusions away from the topic of the paper.
